# Complete genome sequence of *Bacteroides helcogenes* type strain (P 36-108^T^)

**DOI:** 10.4056/sigs.1513795

**Published:** 2011-02-22

**Authors:** Amrita Pati, Sabine Gronow, Ahmet Zeytun, Alla Lapidus, Matt Nolan, Nancy Hammon, Shweta Deshpande, Jan-Fang Cheng, Roxane Tapia, Cliff Han, Lynne Goodwin, Sam Pitluck, Konstantinos Liolios, Ioanna Pagani, Natalia Ivanova, Konstantinos Mavromatis, Amy Chen, Krishna Palaniappan, Miriam Land, Loren Hauser, Yun-Juan Chang, Cynthia D. Jeffries, John C. Detter, Evelyne Brambilla, Manfred Rohde, Markus Göker, Tanja Woyke, James Bristow, Jonathan A. Eisen, Victor Markowitz, Philip Hugenholtz, Nikos C. Kyrpides, Hans-Peter Klenk, Susan Lucas

**Affiliations:** 1 DOE Joint Genome Institute, Walnut Creek, California, USA; 2 DSMZ - German Collection of Microorganisms and Cell Cultures GmbH, Braunschweig, Germany; 3 Los Alamos National Laboratory, Bioscience Division, Los Alamos, New Mexico, USA; 4 Biological Data Management and Technology Center, Lawrence Berkeley National Laboratory, Berkeley, California, USA; 5 Oak Ridge National Laboratory, Oak Ridge, Tennessee, USA; 6 HZI – Helmholtz Centre for Infection Research, Braunschweig, Germany; 7 University of California Davis Genome Center, Davis, California, USA; 8 Australian Centre for Ecogenomics, School of Chemistry and Molecular Biosciences,The University of Queensland, Brisbane, Australia

**Keywords:** strictly anaerobic, mesophilic, nonmotile, Gram-negative, chemoorganotrophic, pig abscess, animal pathogen, *Bacteroidaceae*, GEBA

## Abstract

*Bacteroides helcogenes* Benno *et al.* 1983 is of interest because of its isolated phylogenetic location and, although it has been found in pig feces and is known to be pathogenic for pigs, occurrence of this bacterium is rare and it does not cause significant damage in intensive animal husbandry. The genome of *B. helcogenes* P 36-108^T^ is already the fifth completed and published type strain genome from the genus *Bacteroides* in the family *Bacteroidaceae*. The 3,998,906 bp long genome with its 3,353 protein-coding and 83 RNA genes consists of one circular chromosome and is a part of the *** G****enomic* *** E****ncyclopedia of* *** B****acteria and* *** A****rchaea * project.

## Introduction

Strain P 36-108^T^ (= DSM 20613 = ATCC 35417 = JCM 6297) is the type strain of *Bacteroides helcogenes*, one of currently 39 species in the genus *Bacteroides* [1,2]. The species epithet of *B. helcogenes* is derived from the Greek noun *helkos* meaning ‘abscess’ and the Greek verb *gennaio* meaning ‘produce’, referring to the pathogenic, probably intestinal, abscess-producing properties of the species  [2]. *B. helcogenes* strain P36-108^T^ was isolated from a pig abscess in Japan, and described by Benno *et al*. in 1983 [2]. Nine further isolates of *B. helcogenes* have been obtained from pig abscesses whereas two other isolates originated from pig feces. Here we present a summary classification and a set of features for *B. helcogenes* P 36-108^T^, together with the description of the complete genomic sequencing and annotation.

## Classification and features

A representative genomic 16S rRNA sequence of *B. helcogenes* was compared using NCBI BLAST under default values (e.g., considering only the high-scoring segment pairs (HSPs) from the best 250 hits) with the most recent release of the Greengenes database [3] and the relative frequencies, weighted by BLAST scores, of taxa and keywords (reduced to their stem [4]) were determined. The single most frequent genus was *Bacteroides* (100%) (33 hits in total). Regarding the 21 hits to sequences from other members of the genus, the average identity within HSPs was 92.7%, whereas the average coverage by HSPs was 84.5%. Among all other species, the one yielding the highest score was *Bacteroides ovatus*, which corresponded to an identity of 93.4% and a HSP coverage of 86.6%. The highest-scoring environmental sequence was AM275453 ('fecal microbiota irritable bowel syndrome patients differs significantly from that of healthy subjects'), which showed an identity of 95.5% and a HSP coverage of 84.3%. The most frequently occurring keywords within the labels of environmental samples which yielded hits were 'human' (11.0%), 'fecal' (9.5%), 'microbiota' (8.8%), 'sequenc' (5.4%) and 'gut' (5.4%) (217 hits in total). The most frequently occurring keywords within the labels of environmental samples which yielded hits of a higher score than the highest scoring species were 'fecal/human' (13.3%), 'feedlot' (5.2%), 'bowel, faecal, healthi, irrit, microbiota, patient, significantli, subject, syndrom' (2.7%) and 'beef, cattl, coli, escherichia, feedbunk, habitat, marc, materi, neg, pen, primari, secondari, stec, surfac, synecolog, top, west' (2.6%) (6 hits in total). Most of these keywords are in accordance with the isolation sites of the different isolates and strongly suggest that *B. helcogenes*, like many other species of the genus *Bacteroides*, is associated with the intestinal tract of the host in the case of *B. helcogenes,* this host is the pig [2].

**Figure 1:**
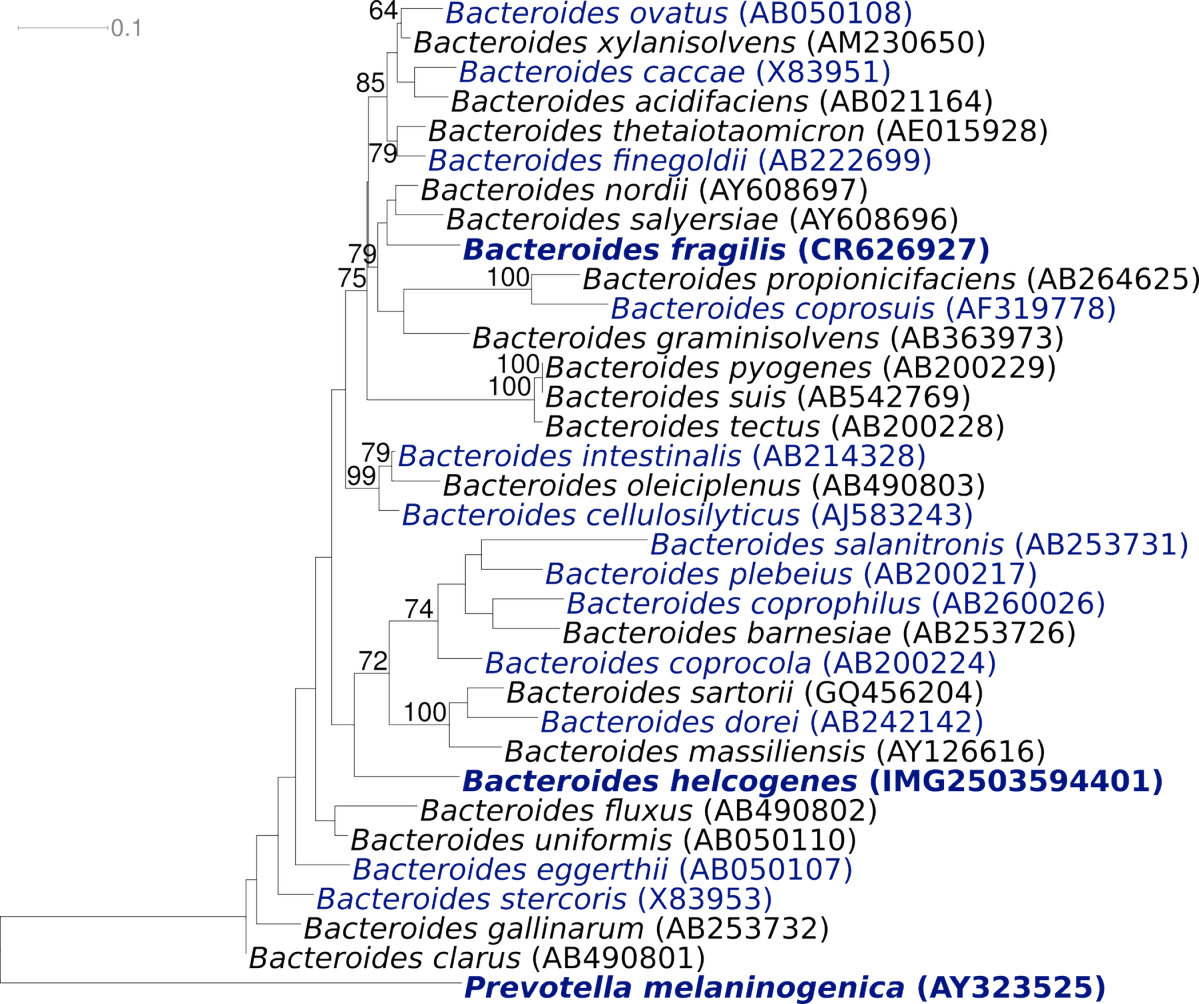
Phylogenetic tree highlighting the position of *B. helcogenes* relative to those type strains within the genus that appeared within a monophyletic *Bacteroides* main clade in preliminary analyses. Note that several of the *Bacteroides* type strain 16S rRNA sequences (from *B. cellulosolvens*, *B. galacturonicus*, *B. pectinophilus*, *B. vulgatus*) did not cluster together with this clade (data not shown, but see [5]) and were omitted from the main phylogenetic inference analysis. The same holds for the sequence from *Anaerorhabdus furcosa* (GU585668; also *Bacteroidaceae*). Other *Bacteroides* species lacked a sufficiently long 16S rRNA sequence and also had to be omitted (*B. coagulans*, *B. xylanolyticus*). The tree was inferred from 1,414 aligned characters [6,7] of the 16S rRNA gene sequence under the maximum likelihood criterion [8] and rooted with the type strain of the family '*Prevotellaceae*'. The branches are scaled in terms of the expected number of substitutions per site. Numbers above branches are support values from 1,000 bootstrap replicates [9] if larger than 60%. Lineages with type strain genome sequencing projects registered in GOLD [10] are shown in blue, published genomes [11] and *Prevotella melaninogenica* released Genbank accession CP002122 in bold.

Figure 1 shows the phylogenetic neighborhood of *B. helcogenes* P 36-108^T^ in a 16S rRNA based tree. The sequences of the five 16S rRNA gene copies in the genome differ from each other by up to 20 nucleotides, and differ by up to 13 nucleotides from the previously published 16S rRNA sequence (AB200227).

**Figure 2:**
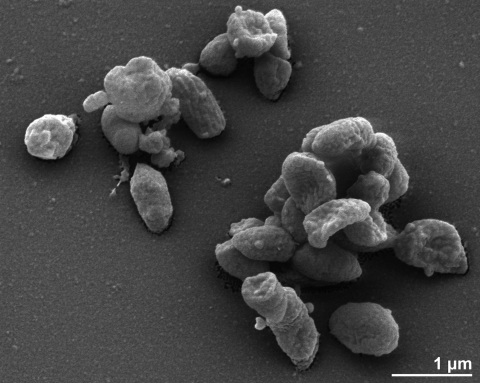
Scanning electron micrograph of *B. helcogenes* P 36-108^T^

The cells of *B. helcogenes* generally have the shape of short rods (0.5-0.6 μm × 0.8-4.0 µm) which occur singly or in pairs (Figure 2). *B. helcogenes* is a Gram-negative, non-pigmented and non spore-forming bacterium (Table 1). The organism is originally described as nonmotile and only five genes associated with motility have been found in the genome (see below). The organism grows well at 37°C but does not grow at 4°C or at 45°C [2]. *B. helcogenes* is strictly anaerobic, chemoorganotrophic and is able to ferment glucose, mannose, fructose, galactose, sucrose, maltose, cellobiose, lactose, xylose, melibiose, raffinose, starch, glycogen, salicin, amygdalin, and xylan [2]. The organism hydrolyzes esculin and starch but does not digest casein, liquify gelatin, reduce nitrate nor produce indole from tryptophan [2]. *B. helcogenes* does not utilize arabinose, ramnose, ribose, trehalose, inulin, glycerol, mannitol, sorbitol, inositol, adonitol, erythritol or gum Arabic [2]. It does not require hemin for growth but does require the presence of CO_2_; it does not show hemolysis. Growth is not enhanced by the addition of 20% bile [2]. Major fermentation products from PYFG broth (peptone yeast extract Fildes glucose broth [26]) are acetic acid and succinic acid; propionic and isobutyric acid are produced in small amounts [2]. *B. helcogenes* is phosphatase, DNase, β-glucuronidase, and glutamic acid decarboxylase active and urease, catalase, lecithinase and lipase inactive [2]. The organism produces ammonium and chondroitin sulfatase [2]. *B. helcogenes* can grow in the presence of kanamycin (1mg/ml), vancomycin (10 µg/ml), colistin (10 µg/ml), erythromycin (60 µg/ml) or polymyxin B (10 µg/ml) but not in the presence of cepharothin (10 µg/ml) or Brilliant green (0.001%) [2].

**Table 1:** Classification and general features of *B. helcogenes* P 36-108^T^ according to the MIGS recommendations [12].

**MIGS ID**	**Property**	**Term**	**Evidence code**
	Current classification	Domain *Bacteria*	TAS [13]
		Phylum *Bacteroidetes*	TAS [14]
		Class '*Bacteroidia*'	TAS [15]
		Order '*Bacteroidales*'	TAS [16]
		Family *Bacteroidaceae*	TAS [17,18]
		Genus *Bacteroides*	TAS [17,19-22]
		Species *Bacteroides helcogenes*	TAS [2,23]
		Type strain P 36-108	TAS [2]
	Gram stain	negative	TAS [2]
	Cell shape	rod-shaped, single or in pairs	TAS [2]
	Motility	non-motile	TAS [2]
	Sporulation	none	TAS [2]
	Temperature range	mesophile	TAS [2]
	Optimum temperature	37°C	TAS [2]
	Salinity	normal	TAS [2]
MIGS-22	Oxygen requirement	strictly anaerobic	TAS [2]
	Carbon source	carbohydrates	TAS [2]
	Energy source	chemoorganotroph	TAS [2]
MIGS-6	Habitat	host	TAS [2]
MIGS-15	Biotic relationship	free-living	TAS [2]
MIGS-14	Pathogenicity	animal pathogen	TAS [2]
	Biosafety level	2	TAS [24]
	Isolation	*Sus scrofa* abscess	TAS [2]
MIGS-4	Geographic location	Japan	TAS [2]
MIGS-5	Sample collection time	1974	TAS [2]
MIGS-4.1	Latitude	not reported	NAS
MIGS-4.2	Longitude	not reported	NAS
MIGS-4.3	Depth	not reported	NAS
MIGS-4.4	Altitude	not reported	NAS

Evidence codes - IDA: Inferred from Direct Assay (first time in publication); TAS: Traceable Author Statement (i.e., a direct report exists in the literature); NAS: Non-traceable Author Statement (i.e., not directly observed for the living, isolated sample, but based on a generally accepted property for the species, or anecdotal evidence). These evidence codes are from of the Gene Ontology project [25]. If the evidence code is IDA, then the property was directly observed by one of the authors or an expert mentioned in the acknowledgements.

### Chemotaxonomy

Little chemotaxonomic information is available for strain P 36-108^T^. Thus far, only the fatty acid composition has been elucidated. The major fatty acids found (>10%) were *anteiso-*C_15:0_, C_15:0_ and *iso-*C_15:0.3-OH_. Also, *iso-*C_15:0_, C_16:0_, and *cis* C_18:1_ were detected in a proportion ranging between 5% to 10% of the total fatty acids (unpublished data).

## Genome sequencing and annotation

### Genome project history

This organism was selected for sequencing on the basis of its phylogenetic position [27], and is part of the *** G****enomic* *** E****ncyclopedia of* *** B****acteria and* *** A****rchaea * project [28]. The genome project is deposited in the Genomes OnLine Database [10] and the complete genome sequence is deposited in GenBank. Sequencing, finishing and annotation were performed by the DOE Joint Genome Institute (JGI). A summary of the project information is shown in Table 2.

**Table 2:** Genome sequencing project information

**MIGS ID**	**Property**	**Term**
MIGS-31	Finishing quality	Finished
MIGS-28	Libraries used	Three genomic libraries: one 454 pyrosequence standard library, one 454 PE library (9 kb insert size), one Illumina library
MIGS-29	Sequencing platforms	Illumina GAii, 454 GS FLX Titanium
MIGS-31.2	Sequencing coverage	56.3 × Illumina; 36.7 × pyrosequence
MIGS-30	Assemblers	Newbler version 2.3-PreRelease-10-21-2009-gcc-4.1.2-threads, Velvet, phrap
MIGS-32	Gene calling method	Prodigal 1.4, GenePRIMP
	INSDC ID	CP002352
	Genbank Date of Release	January 18, 2011
	GOLD ID	Gc01593
	NCBI project ID	41913
	Database: IMG-GEBA	2503538016
MIGS-13	Source material identifier	DSM 20613
	Project relevance	Tree of Life, GEBA

### Growth conditions and DNA isolation

*B. helcogenes* P 36-108^T^, DSM 20613, was grown anaerobically in medium 104 (PYG Medium) [29] at 37°C. DNA was isolated from 0.5-1 g of cell paste using MasterPure Gram-positive DNA purification kit (Epicentre MGP04100) following the standard protocol as recommended by the manufacturer, with modification st/DL for cell lysis as described in Wu *et al*. [28]. DNA is available through the DNA Bank Network [30,31].

### Genome sequencing and assembly

The genome was sequenced using a combination of Illumina and 454 sequencing platforms. All general aspects of library construction and sequencing can be found at the JGI website [32]. Pyrosequencing reads were assembled using the Newbler assembler version 2.3-PreRelease-10-21-2009-gcc-4.1.2-threads (Roche). The initial Newbler assembly consisting of 48 contigs in two scaffolds was converted into a phrap assembly by [33] making fake reads from the consensus, to collect the read pairs in the 454 paired end library. Illumina GAii sequencing data (225.3 Mb) was assembled with Velvet [34] and the consensus sequences were shredded into 1.5 kb overlapped fake reads and assembled together with the 454 data. The 454 draft assembly was based on 146.7 Mb 454 draft data and all of the 454 paired end data. Newbler parameters are -consed -a 50 -l 350 -g -m -ml 20. The Phred/Phrap/Consed software package [33] was used for sequence assembly and quality assessment in the subsequent finishing process. After the shotgun stage, reads were assembled with parallel phrap (High Performance Software, LLC). Possible mis-assemblies were corrected with gapResolution [32], Dupfinisher [35], or sequencing cloned bridging PCR fragments with subcloning or transposon bombing (Epicentre Biotechnologies, Madison, WI). Gaps between contigs were closed by editing in Consed, by PCR and by Bubble PCR primer walks (J.-F.Chang, unpublished). A total of 160 additional reactions and 4 shatter libraries were necessary to close gaps and to raise the quality of the finished sequence. Illumina reads were also used to correct potential base errors and increase consensus quality using a software Polisher developed at JGI [36]. The error rate of the completed genome sequence is less than 1 in 100,000. Together, the combination of the Illumina and 454 sequencing platforms provided 93 × coverage of the genome. The final assembly contained 500,148 pyrosequence and 6,257,254 Illumina reads.

### Genome annotation

Genes were identified using Prodigal [37] as part of the Oak Ridge National Laboratory genome annotation pipeline, followed by a round of manual curation using the JGI GenePRIMP pipeline [38]. The predicted CDSs were translated and used to search the National Center for Biotechnology Information (NCBI) nonredundant database, UniProt, TIGR-Fam, Pfam, PRIAM, KEGG, COG, and InterPro databases. Additional gene prediction analysis and functional annotation was performed within the Integrated Microbial Genomes - Expert Review (IMG-ER) platform [39].

## Genome properties

**Figure 3:**
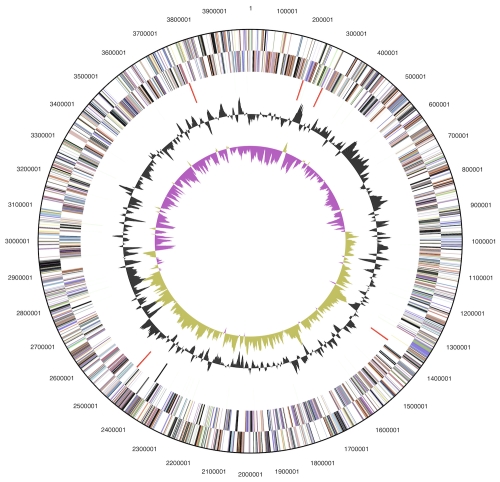
Graphical circular map of the chromosome. From outside to the center: Genes on forward strand (color by COG categories), Genes on reverse strand (color by COG categories), RNA genes (tRNAs green, rRNAs red, other RNAs black), GC content, GC skew.

The genome consists of a 3,998,906 bp long chromosome with a GC content of 44.7% (Table 3 and Figure 3). Of the 3,436 genes predicted, 3,353 were protein-coding genes, and 83 RNAs; 109 pseudogenes were also identified. The majority of the protein-coding genes (64.5%) were assigned with a putative function while the remaining ones were annotated as hypothetical proteins. The distribution of genes into COGs functional categories is presented in Table 4.

**Table 3:** Genome Statistics

**Attribute**	**Value**	**% of Total**
Genome size (bp)	3,998,906	100.00%
DNA coding region (bp)	3,583,947	89.62%
DNA G+C content (bp)	1,788,209	44.72%
Number of replicons	1	100.00%
Extrachromosomal elements	0	
Total genes	3,436	100.00%
RNA genes	83	2.42%
rRNA operons	5	
Protein-coding genes	3,353	97.58%
Pseudo genes	109	3.17%
Genes with function prediction	2,215	64.46%
Genes in paralog clusters	454	13.21%
Genes assigned to COGs	2103	61.20%
Genes assigned Pfam domains	2360	68.68%
Genes with signal peptides	980	28.52%
Genes with transmembrane helices	798	23.22%
CRISPR repeats	1	

**Table 4:** Number of genes associated with the general COG functional categories

**Code**	**value**	**%age**	**Description**
J	147	6.5	Translation, ribosomal structure and biogenesis
A	0	0	RNA processing and modification
K	157	6.9	Transcription
L	125	5.5	Replication, recombination and repair
B	0	0	Chromatin structure and dynamics
D	20	0.9	Cell cycle control, cell division, chromosome partitioning
Y	0	0	Nuclear structure
V	67	2.9	Defense mechanisms
T	125	5.5	Signal transduction mechanisms
M	245	10.8	Cell wall/membrane/envelope biogenesis
N	5	0.2	Cell motility
Z	0	0	Cytoskeleton
W	0	0	Extracellular structures
U	48	2.1	Intracellular trafficking, secretion, and vesicular transport
O	66	2.9	Posttranslational modification, protein turnover, chaperones
C	120	5.3	Energy production and conversion
G	185	8.1	Carbohydrate transport and metabolism
E	149	6.5	Amino acid transport and metabolism
F	67	2.9	Nucleotide transport and metabolism
H	120	5.3	Coenzyme transport and metabolism
I	64	2.8	Lipid transport and metabolism
P	161	7.6	Inorganic ion transport and metabolism
Q	20	0.9	Secondary metabolites biosynthesis, transport and catabolism
R	266	11.7	General function prediction only
S	122	5.4	Function unknown
-	1,333	38.8	Not in COGs
